# Short-Term Physiological Effects of Moderate PEEP Levels in Invasively Ventilated Patients Without Acute or Chronic Lung Disease

**DOI:** 10.3390/medsci14020168

**Published:** 2026-03-27

**Authors:** Camila Vantini Capasso Palamim, Fernando Augusto Lima Marson

**Affiliations:** 1 Laboratory of Molecular Biology and Genetics, Postgraduate Program of Health Sciences, Postgraduate Program in Health Data Science, São Francisco University (Universidade São Francisco—USF), Bragança Paulista 12916-900, SP, Brazil; 2 Laboratory of Clinical and Molecular Microbiology, Postgraduate Program of Health Sciences, São Francisco University (Universidade São Francisco—USF), Bragança Paulista 12916-900, SP, Brazil; 3 LunGuardian Research Group—Epidemiology of Respiratory and Infectious Diseases, Postgraduate Program of Health Sciences, São Francisco University (Universidade São Francisco—USF), Bragança Paulista 12916-900, SP, Brazil

**Keywords:** driving pressure, gas exchange, hemodynamics, mechanical ventilation, oxygenation index, positive end-expiratory pressure

## Abstract

**Background/Objectives:** Positive end-expiratory pressure (PEEP) is a standardized component of the invasive mechanical ventilation (IMV) settings to improve oxygenation; however, its physiological effects in patients with no documented prior lung disease remain poorly defined. This study evaluated the impact of moderate PEEP variations on macrohemodynamic parameters, gas exchange, and driving pressure (ΔP). **Methods:** This single-arm, non-randomized, crossover study included adult intensive care unit (ICU) patients with no documented prior lung disease during the early phase of IMV. Sequential PEEP levels of 6, 8, and 10 cmH_2_O were applied for 30 min each within the first 24 h of ICU admission, while all other ventilatory parameters were kept constant. Arterial blood gases [partial pressure of oxygen (PaO_2_), partial pressure of carbon dioxide (PaCO_2_), and arterial oxygen saturation (SaO_2_)], oxygenation index [PaO_2_/fraction of inspired oxygen (FiO_2_)], systolic, diastolic, and mean arterial pressures, ΔP, and static compliance (Cstat) were measured. Friedman and Mann–Whitney U tests were used, with adjustment for multiple comparisons. **Results:** A total of 150 patients were enrolled (64.7% male). The observed mortality rate was 53.3%; however, mortality was not defined as a primary or secondary outcome, and was used only as a grouping variable for comparative analyses. Intraindividual comparison across PEEP levels of 6, 8, and 10 cmH_2_O showed small but significant reductions in systolic and mean arterial pressure at higher PEEP (*p*-value < 0.05), with Bonferroni-adjusted significance for PEEP 6 vs. 10. No significant differences were observed in oxygenation (SaO_2_, PaO_2_, and PaO_2_/FiO_2_), PaCO_2_, ΔP, or Cstat. These results suggest that moderate PEEP changes produced limited macrohemodynamic effects without relevant impact on gas exchange or respiratory mechanics. Overall, no clinically relevant or statistically significant differences were observed in gas exchange, macrohemodynamic parameters, ΔP, or Cstat across PEEP levels when mortality was used as the grouping variable. Among survivors, higher PEEP was associated with modest reductions in systolic and mean arterial pressures and higher PaCO_2_ values; however, these findings did not translate into consistent physiological benefits. **Conclusions:** In mechanically ventilated patients with no documented prior lung disease, PEEP may exert divergent effects on macrohemodynamics, gas exchange, and ΔP, supporting a cautious and individualized approach to PEEP selection in this population.

## 1. Introduction

One of the major challenges in the clinical practice of mechanical ventilation (MV) management is understanding the interaction between ventilator-delivered parameters and how lung tissue accommodates and responds to them. These interactions are mainly determined by two factors: (i) ventilator settings defined by the operator, such as tidal volume, inspiratory and expiratory pressures, respiratory flow, and respiratory rate; and (ii) the structural and functional condition of the lung parenchyma, which may impair gas exchange due to increased heterogeneity, alveolar collapse, and regional hyperdistention [1].

The adoption of lung-protective strategies has been shown to reduce the risk of ventilator-induced lung injury (VILI) in patients receiving positive pressure ventilation, particularly in populations with acute lung injury such as acute respiratory distress syndrome (ARDS) and pneumonia [2]. However, patients undergoing MV for extrapulmonary reasons (e.g., neurological impairment or trauma) often present different lung mechanics and hemodynamic responses. In this subgroup, the optimal way to individualize ventilatory parameters to maximize functional benefit while minimizing lung injury remains less clearly defined.

Among ventilatory parameters, positive end-expiratory pressure (PEEP) plays a central role in maintaining alveolar stability at end expiration and improving oxygenation. Increasing PEEP can expand the effective alveolar surface area available for gas exchange, while improved alveolar stability may reduce the thickness of the alveolar–capillary membrane, facilitating gas diffusion and improving arterial oxygen partial pressure (PaO_2_) and arterial oxygen saturation (SaO_2_) [3,4]. In clinical practice, PEEP promotes the recruitment of unstable or collapsed alveoli, improves gas exchange and tissue oxygenation, and mitigates the heterogeneity of mechanical stresses distribution across the lung parenchyma [5,6].

However, PEEP may either optimize or impair pulmonary function depending on the underlying disease, chest wall characteristics, hemodynamic condition, and individual physiological response. This dual and context-dependent effect reinforces that PEEP selection should not rely on a rigid interpretation of isolated lung mechanics. Instead, it highlights the need for integrated and clinically meaningful markers to guide individualized PEEP titration. In this context, respiratory system compliance can contribute to clinical assessment but should be interpreted alongside the broader clinical picture rather than used as the sole determinant of PEEP selection [7].

Compliance reflects the elastic properties of the respiratory system and is assessed by measuring the lung’s ability to expand in response to applied pressure [8]. Conversely, PEEP levels that exceed the lung’s recruitment potential or applied in patients with low recruitability may produce unfavorable physiological effects, including reduced venous return, decreased cardiac output, and potential impairment of right ventricular performance. In recruitable lungs, such as in selected patients with ARDS, PEEP may reduce pulmonary vascular resistance by promoting alveolar recruitment and attenuating hypoxic pulmonary vasoconstriction, thereby supporting right ventricular function and improving gas exchange. Thus, the hemodynamic and oxygenation effects of PEEP are context-dependent and should be interpreted according to underlying disease and patient physiology rather than fixed thresholds [9].

Driving pressure (ΔP), defined as the difference between plateau pressure (Pplat) and PEEP, is directly influenced by PEEP adjustments. When PEEP leads to effective alveolar recruitment and improved respiratory system compliance, ΔP may decrease due to a reduction in Pplat. Elevated ΔP has been identified as an independent predictor of mortality and although consensus is lacking, studies suggest maintaining ΔP values below 15 cmH_2_O in patients with ARDS [10,11].

Most studies on PEEP have been conducted in patients with ARDS, focusing on its potential role in lung protection [12,13]. In this context, PEEP is applied to maintain alveolar recruitment, thereby reducing VILI, improving gas exchange, and enhancing oxygenation [14]. Beyond ARDS, other conditions may substantially modify lung mechanics and the response to MV. Chronic obstructive pulmonary disease, for example, is characterized by severe airflow limitation, gas trapping, dynamic hyperinflation, and intrinsic PEEP, which impair ventilatory efficiency [15]. Similarly, obesity alters respiratory anatomy and physiology, complicating airway management, ventilator settings, and patient–ventilator interaction during MV [16,17,18]. Nevertheless, studies specifically addressing the effects of PEEP in patients without preexisting lung disease remain scarce [16], and conclusions are often extrapolated from post hoc analyses of patients with prior lung injury.

Although optimal PEEP titration is well established for ARDS management, the appropriate strategy for patients without ARDS remains controversial due to heterogeneous and sometimes conflicting data [19]. Recent evidence suggests that higher PEEP levels in this population do not consistently translate into survival benefit and may increase the need for hemodynamic support [20,21]. Furthermore, the recognized relationship between PEEP and mechanical power reinforces the importance of cautious titration, as excessive levels may contribute to unnecessary lung stress and strain [22]. Consequently, more conservative PEEP approaches have been proposed to preserve hemodynamic stability while ensuring adequate ventilatory support.

Given the clinical importance of PEEP, the present study aimed to evaluate the physiological response to controlled PEEP variation in adult patients without documented prior lung disease and with preserved global respiratory system compliance during the early phase of invasive mechanical ventilation (IMV, <24 h) in a university hospital setting. The analysis focused on routinely monitored variables, including macrohemodynamic parameters, gas exchange, and ΔP, under controlled ventilatory conditions in which all other ventilator settings were kept constant to isolate the effect of three moderate PEEP adjustments (6, 8, and 10 cmH_2_O).

## 2. Materials and Methods

### 2.1. Study Design and Setting

The single-arm, non-randomized, cross-over study evaluated adult patients admitted during the year of 2024 to the intensive care unit (ICU) of the São Francisco de Assis University Hospital, in Bragança Paulista, São Paulo, Brazil, who required IMV. The general characteristics of the ICU have been previously described in the literature [23].

The study was conducted in accordance with the Declaration of Helsinki, and was approved by the Institution’s Ethics Committee (Submission no. 29718820.9.0000.5514, date of approval: 30 March 2020; Study approval no. 3.939.784, date of approval: 29 May 2020). Informed consent was obtained from all subjects involved in the study.

### 2.2. Patient Selection and Definition of Lung Health

Inclusion and exclusion criteria were assessed by a multidisciplinary team responsible for patient follow-up at our institution. Lung healthy status was defined by the absence of confirmed clinical disease rather than by the absence of risk factors such as tobacco exposure.

Eligible participants were adults (≥18 years), either medical or surgical patients. All individuals were enrolled within the first 24 h after initiation of MV.

For the purposes of this study, the cause of hospitalization and the reason for ICU admission were identical, as all patients were admitted to the ICU as part of their initial acute management or immediate postoperative care.

Only patients without documented prior lung disease were included. A history of chronic obstructive pulmonary disease, cystic fibrosis, asthma, chronic bronchitis, lung neoplasms, or interstitial lung disease was excluded based on a thorough review of medical records and structured family interviews.

All participants also underwent a detailed clinical evaluation by a physician, including physical examination and assessment of respiratory symptoms, to identify any underlying pulmonary conditions (such as pneumonia, acute hypoxemic respiratory failure, or other primary respiratory causes) that could confound the study outcomes.

Additional exclusion criteria included hemodynamic instability, pneumothorax, undrained pleural effusion, lack of peripheral arterial access, previous MV, or sepsis of pulmonary origin recorded as the reason for hospitalization. Patients presenting with clinically significant thoracic injuries that could markedly alter chest wall mechanics, such as flail chest, were also excluded.

### 2.3. Clinical and Demographic Data Collection

The following data were collected: sex (male or female), age (years), primary diagnosis (as determined by the multidisciplinary team), length of hospital stay (days), duration of IMV (days), medical history (previous comorbidities), height (cm), body mass index (BMI, kg/m^2^), presence of ventilator-associated pneumonia, requirement for tracheostomy, clinical outcome (hospital discharge or death), and type of ventilatory support. Clinical outcomes and duration of IMV were collected as follow-up variables for descriptive and contextual purposes.

Ventilator-associated pneumonia was defined according to standard criteria as pneumonia occurring ≥ 48 h after the start of MV and was recorded only when diagnosed during the ICU stay after study inclusion.

The primary objective of the protocol was restricted to the short-term physiological effects of different PEEP settings on macrohemodynamic parameters, gas exchange, and ΔP under controlled ventilatory conditions. Accordingly, outcome and IMV duration data were analyzed only as complementary follow-up information and were not included in the main effect analyses.

### 2.4. PEEP Intervention Protocol

The study evaluate the impact of different PEEP levels (6, 8, and 10 cmH_2_O) applied to the same patient on PaO_2_, arterial carbon dioxide partial pressure (PaCO_2_), SaO_2_, oxygenation index (PaO_2_/FiO_2_; defined as the ratio between PaO_2_ and the fraction of inspired oxygen), systolic arterial pressure, diastolic arterial pressure, mean arterial pressure [assessed by invasive blood pressure (IBP; DX-2020, Dixtal Biomédica, Santo Amaro, São Paulo, Brazil) monitoring], ΔP, and static compliance (Cstat). Arterial blood gases (PaO_2_, PaCO_2_, and SaO_2_) were obtained from peripheral arterial samples collected by a nurse at the end of each PEEP level.

Macrohemodynamic instability was defined as heart rate > 120 b.p.m. or mean arterial pressure < 65 mmHg. Oxygen desaturation (SaO_2_ < 90%) was monitored separately as a respiratory safety criterion during ventilatory adjustments. The study protocol was interrupted if hemodynamic instability developed during data collection.

Each PEEP level was maintained for 30 min before applying the subsequent level, based on physiological evidence indicating that this interval is sufficient for lung tissue stress relaxation and for the establishment of a steady state in respiratory mechanics and gas exchange, particularly carbon dioxide (CO_2_) elimination, following ventilatory setting changes [16,24].

Measurements of physiological and biochemical markers were obtained at the three PEEP levels within the first 24 h of ICU admission. The use of this standardized stabilization period aimed to ensure that recorded measurements reflected the true physiological response to the applied PEEP level while minimizing the influence of transient cardiorespiratory fluctuations immediately after ventilator adjustments.

In accordance with the study protocol, PEEP levels were capped at 10 cmH_2_O to maintain a conservative and standardized ventilatory range for this population [25]. No prior lung recruitment maneuvers were performed.

All patients were sedated to achieve a Richmond Agitation-Sedation Scale score of -5 and were receiving controlled MV. Neuromuscular blocking agents were not administered during the intervention period.

### 2.5. Ventilator Settings

The FiO_2_ was fixed—titrated to maintain an SaO_2_ above 90% and kept constant during data collection.

Tidal volume was set between 6 and 8 mL/kg of predicted body weight, with height estimated by the hospital nutrition team. Plateau pressure (Pplat) was maintained below 30 cmH_2_O, and respiratory rate was adjusted to maintain pH (potential of hydrogen) above 7.20.

The FiO_2_ values applied in our protocol ranged from 21% to 30%, which are considered low according to the literature, to achieve the target SaO_2_.

Static compliance (Cstat) was calculated as the tidal volume divided by the difference between Pplat and PEEP, with Pplat measured after a 2 s inspiratory pause.

Hemoglobin concentration was measured at all PEEP levels and remained above 9 g/dL in all patients.

Following the intervention, MV settings were returned to their initial values.

### 2.6. Statistical Analysis

Statistical analyses were performed using IBM SPSS (Statistical Package for the Social Sciences) Statistics for Macintosh, Version 27.0 (IBM Corp., Armonk, NY, USA). Descriptive data are presented as mean ± standard deviation or as absolute (N) and relative (%) frequencies. The normality of continuous variables was assessed using a combination of approaches: (i) descriptive measures of central tendency; (ii) graphical methods, including normal Q–Q (Quantile–Quantile) plots, detrended Q–Q plots, and boxplots; and (iii) statistical tests for normality, specifically the Kolmogorov–Smirnov and Shapiro–Wilk tests.

Inferential analyses were conducted using the Friedman test to evaluate differences in dependent markers across multiple PEEP levels, considering grouping by clinical outcomes. For comparisons between two independent groups, the Mann–Whitney U test was applied. Categorical variables were analyzed using the chi-square test or Fisher’s exact test, as appropriate. The *p*-values for multiple comparisons were adjusted using the false discovery rate or Bonferroni correction methods.

Dependent variables included PaO_2_, PaCO_2_, SaO_2_, oxygenation index, systolic arterial pressure, diastolic arterial pressure, mean arterial pressure, ΔP, and Cstat. Comparisons according to hospital outcome were performed for descriptive purposes only and were not part of the primary study objectives.

Sample size estimation was performed using G*Power software, version 3.1.9.6 [26], with a significance level (α) set at 0.05 for all analyses.

## 3. Results

We included 150 patients in the study, of whom 97 (64.7%) were men and 53 (35.3%) were women; 98 patients (65.3%) were admitted for surgical reasons. The main causes of hospitalization were elective surgery (41; 27.3%), polytrauma (37; 24.7%), traumatic brain injury (30; 20.0%), and non-pulmonary sepsis (23; 15.3%) (Table 1).

The most frequent comorbidities were systemic arterial hypertension (52; 34.7%), diabetes mellitus (32; 21.3%), smoking and cardiopathy (27; 18% each), and alcohol use (25; 16.7%). The predominant mode of MV was volume-controlled ventilation (138; 92.0%), followed by pressure-controlled ventilation (12; 8.0%). During ICU follow-up, 47 patients (31.1%) developed ventilator-associated pneumonia and 59 (39.3%) required tracheostomy (Table 1). Eighty patients (53.3%) died (Table 1).

Dependent variables were compared according to PEEP levels within the same patient. All other MV parameters were maintained constant during the intervention and adjusted individually according to the 2013 Brazilian MV Guidelines [27].

All primary physiological analyses were performed using the full cohort (Table 2). Subgroup comparisons according to hospital outcome (death vs. discharge) were conducted only as exploratory descriptive analyses and were not defined as study outcomes (Table 3).

The intraindividual analysis of physiological variables across the three PEEP levels demonstrated statistically significant effects only on macrohemodynamic arterial pressure parameters, with no relevant impact on gas exchange or respiratory mechanics variables.

A small but statistically significant reduction in systolic arterial pressure was observed with increasing PEEP levels (129.25 ± 26.64 mmHg at PEEP 6; 129.01 ± 27.04 mmHg at PEEP 8; 126.43 ± 25.45 mmHg at PEEP 10; *p*-value = 0.042). A similar pattern was found for mean arterial pressure (85.39 ± 14.51; 85.85 ± 15.23; 84.25 ± 14.52 mmHg, respectively; *p*-value = 0.029). After adjustment for multiple comparisons using the Bonferroni correction, statistical significance was maintained specifically for the comparison between PEEP 6 and PEEP 10. Diastolic arterial pressure did not differ significantly across PEEP levels (*p*-value = 0.153).

Regarding oxygenation parameters, no significant differences were detected between PEEP levels. Peripheral oxygen saturation (SaO_2_) remained stable (97.79 ± 2.29%; 97.73 ± 2.31%; 97.78 ± 2.22%; *p*-value = 0.649), as did PaO_2_ (121.39 ± 36.96 mmHg; 118.97 ± 34.49 mmHg; 120.47 ± 38.11 mmHg; *p*-value = 0.716) and the PaO_2_/FiO_2_ ratio (373.16 ± 140.50; 364.90 ± 127.60; 365.27 ± 128.46; *p*-value = 0.844). PaCO_2_ showed a slight upward trend at higher PEEP levels, but without statistical significance (41.62 ± 6.87 mmHg; 41.87 ± 6.78 mmHg; 42.54 ± 6.45 mmHg; *p*-value = 0.073).

No significant differences were observed in respiratory mechanics variables. Driving pressure (ΔP) remained unchanged across PEEP levels (10.13 ± 3.19 cmH_2_O; 9.89 ± 3.11 cmH_2_O; 9.99 ± 3.44 cmH_2_O; *p*-value = 0.415), as did Cstat of the respiratory system (46.80 ± 17.75 mL/cmH_2_O; 48.30 ± 20.16 mL/cmH_2_O; 49.32 ± 23.50 mL/cmH_2_O; *p*-value = 0.415). Overall, these findings indicate that moderate PEEP variations were associated with modest macrohemodynamic changes, without significant effects on gas exchange or ventilatory mechanics in this cohort.

The association between PEEP and the evaluated markers—namely, PaO_2_, PaCO_2_, SaO_2_, oxygenation index, systolic arterial pressure, diastolic arterial pressure, mean arterial pressure, ΔP, and Cstat—according to clinical outcomes (hospital discharge or death) is presented in Table 3. When analyzing markers related to macrohemodynamics, gas exchange, and ΔP, no statistically significant changes were observed in most dependent variables across the different PEEP levels.

However, patients who experienced clinical recovery after the intervention exhibited lower systolic and mean arterial pressures at a PEEP of 10 cmH_2_O, as well as higher PaCO_2_ values at the same PEEP level. Furthermore, after correction for multiple comparisons, some nondependent markers showed statistically significant differences among PEEP levels, even when stratified by clinical outcomes.

**Figure 1:**
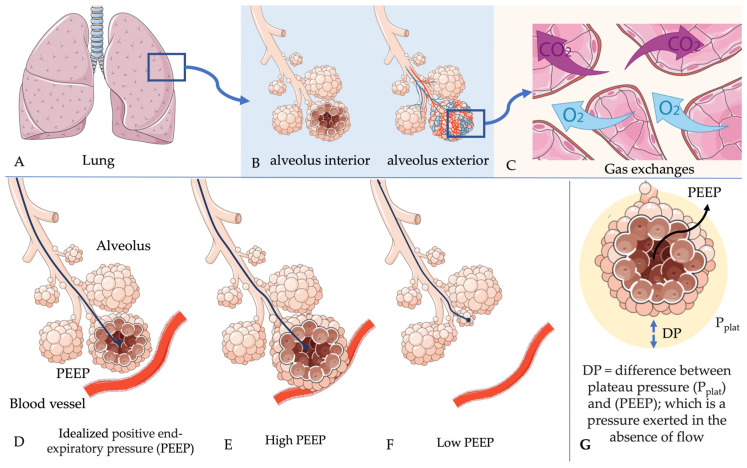
Morphological and physiological aspects of the lung related to gas exchange, positive end-expiratory pressure (PEEP), and driving pressure (DP or ΔP). (**A**) Anatomical representation of the lung and its pulmonary lobes. (**B**) Schematic of alveolar structure: on the left, alveoli filled with air; on the right, alveoli surrounded by blood vessels. (**C**) Illustration of gas exchange by simple diffusion, where oxygen (O_2_) and carbon dioxide (CO_2_) move across the alveolar–capillary interface. (**D**) Ideal conditions for gas exchange, emphasizing a large surface area and minimal thickness of the blood-gas barrier, in accordance with Fick’s law. (**E**) Impaired gas exchange due to PEEP levels that exceed recruitment capacity, causing alveolar compression of adjacent blood vessels. (**F**) Impaired gas exchange due to insufficient PEEP, increasing the distance between alveoli and blood vessels. (**G**) Driving pressure (ΔP), defined as the difference between plateau pressure (Pplat) and PEEP under end-inspiratory no-flow conditions (ΔP = Pplat − PEEP). Plateau pressure (Pplat) is illustrated outside the alveolus to emphasize that it is an airway pressure measured at the ventilator during an inspiratory pause, representing the static elastic recoil of the respiratory system, rather than a directly measured intra-alveolar or pleural pressure.

Figure 1 illustrates, in an integrated manner, the main morphological and physiological aspects of the lung related to gas exchange and the influence of MV, particularly PEEP and ΔP. Initially, the anatomical organization of the lung and its pulmonary lobes is presented, providing context for the structural complexity of the organ responsible for ventilation and perfusion. Subsequently, a schematic representation of the alveoli highlights the close spatial relationship between air spaces and blood vessels, a fundamental condition for efficient gas exchange. The process of simple diffusion of oxygen and carbon dioxide across the alveolar–capillary interface is emphasized as the central mechanism of gas exchange, directly dependent on the available surface area and the thickness of the blood–gas barrier, in accordance with Fick’s law. The figure also demonstrates how inappropriate ventilatory conditions can disrupt this balance: PEEP levels that exceed recruitment capacity may lead to compression of adjacent capillaries, thereby reducing perfusion, whereas insufficient PEEP increases the functional distance between alveoli and blood vessels, impairing gas diffusion. Finally, ΔP is depicted as the difference between Pplat and PEEP, representing the pressure effectively applied to the alveolar walls in the absence of airflow, a key determinant of pulmonary mechanical stress during MV.

Arterial pH was monitored as part of routine ICU management and remained within clinically acceptable ranges throughout the protocol, showing no systematic variation related to short-term PEEP adjustments; therefore, it was not included among the primary analytical variables.

**Figure 2:**
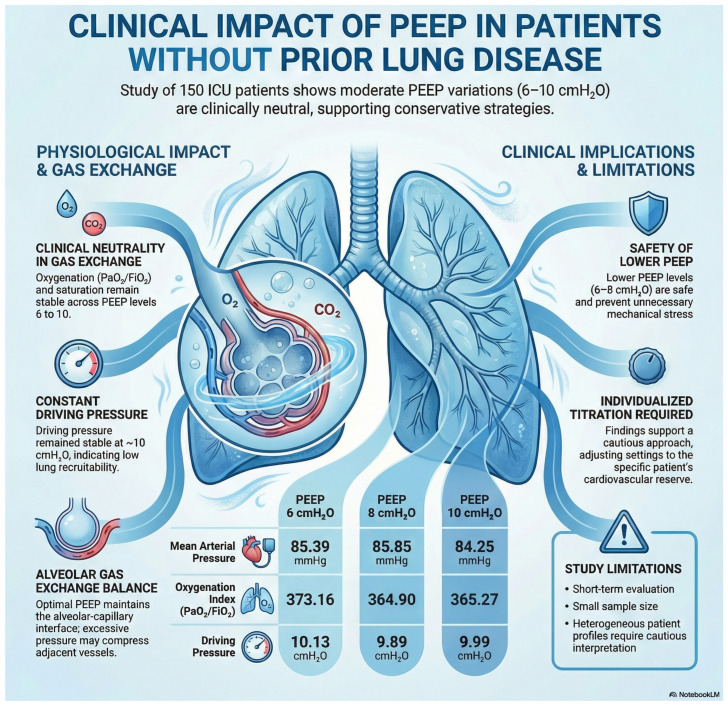
Physiological and clinical effects of moderate positive end-expiratory pressure (PEEP) variation (6, 8, and 10 cmH_2_O) in invasively ventilated patients without evidence of chronic pulmonary dysfunction. The infographic summarizes the intraindividual responses to controlled PEEP adjustments during the early phase of invasive mechanical ventilation. Mean arterial pressure (MAP), oxygenation index (PaO_2_/FiO_2_), and driving pressure (ΔP) remained stable across PEEP levels, with only small fluctuations that did not reach clinical relevance. PEEP of 6 cmH_2_O was associated with MAP of 85.39 mmHg, PaO_2_/FiO_2_ of 373.16, and ΔP of 10.13 cmH_2_O; PEEP of 8 cmH_2_O with MAP of 85.85 mmHg, PaO_2_/FiO_2_ of 364.90, and ΔP of 9.89 cmH_2_O; and PEEP of 10 cmH_2_O with MAP of 84.25 mmHg, PaO_2_/FiO_2_ of 365.27, and ΔP of 9.99 cmH_2_O. The schematic representation highlights the physiological rationale underlying these findings: in patients with preserved global respiratory system compliance, moderate PEEP adjustments do not substantially modify alveolar recruitment or the alveolar–capillary interface, resulting in a minimal impact on gas exchange or mechanical stress. The figure also summarizes the clinical implications of these results, emphasizing the safety of lower PEEP levels (6–8 cmH_2_O), the limited benefit of routine escalation to 10 cmH_2_O, and the importance of individualized titration based on hemodynamic reserve and lung recruitability. Study limitations, including short-term assessment, heterogeneous clinical profiles, and absence of advanced hemodynamic monitoring, are also noted. FiO_2_, fraction of inspired oxygen; PaO_2_, arterial oxygen partial pressure. NotebookLM (Google LLC) was used under an institutional license provided by the University of São Francisco (Universidade São Francisco—USF) through Google Workspace for Education. This license allowed authorized faculty and students to use the tool for academic and research purposes. The platform was used to support the organization and analysis of academic materials. Data usage followed institutional policies for privacy, security, and responsible use of digital tools.

To complement the numerical analyses, Figure 2 provides an integrated visualization of the physiological responses to PEEP variation in this cohort. As illustrated, mean arterial pressure, oxygenation index, and ΔP exhibited only minimal fluctuations across PEEP levels of 6, 8, and 10 cmH_2_O, reinforcing the statistical findings that moderate PEEP adjustments did not meaningfully alter macrohemodynamics, gas exchange, or respiratory mechanics. The graphical comparison demonstrates that ΔP remained consistently around 10 cmH_2_O, suggesting low recruitability and preserved compliance in this population. Similarly, PaO_2_/FiO_2_ ratios showed negligible variation, supporting the interpretation that alveolar recruitment was not substantially modified by the applied PEEP range. Together, these visual and analytical results indicate that, in patients without documented prior lung disease, moderate PEEP changes exert limited short-term physiological effects, underscoring the importance of individualized rather than protocolized PEEP titration.

## 4. Discussion

Data from 150 participants were analyzed, revealing a predominance of male patients admitted to the ICU after surgery, with polytrauma being a common cause of hospitalization. These findings reflect the profile of the study center, which serves as a regional referral hospital for trauma [23].

Regarding PEEP, the literature recommends its use to mitigate the effects of orotracheal intubation, which can result in loss of lung volume and reduced functional residual capacity [28]. In clinical practice, a PEEP of approximately 8 cmH_2_O is commonly applied as a preventive strategy in this context. However, lower PEEP levels (around 5 cmH_2_O) has also been reported safe in patients requiring orotracheal intubation for reasons unrelated to chronic lung disease, contributing to the prevention of VILI [28,29,30].

In the present study, practically none of the evaluated markers showed significant responsiveness to the tested PEEP levels. Macrohemodynamic parameters, gas exchange indices, and ΔP values remained stable across the different PEEP settings.

Our study focused on ICU patients with no documented prior lung disease and without a diagnosis of ARDS, of either medical or surgical origin, aiming to evaluate the influence of PEEP in this population. PEEP is an integral component of IMV, primarily used to maintain end-expiratory lung volume and reduce alveolar collapse, with potential effects on oxygenation and respiratory mechanics; however, its titration should be individualized according to patient physiology and clinical context. Studies investigating increased PEEP during abdominal and thoracic surgeries have shown improvements in oxygenation, although these studies did not assess whether such improvements persisted into the postoperative period [25,31,32,33].

The pressures generated by positive inspiratory and expiratory pressures during IMV directly affect right and left ventricular function and may result in macrohemodynamic parameters consequences such as reductions in arterial pressure and alterations in heart rate [34]. Lung exposure to positive pressure alters lung volumes, significantly modifying pulmonary vascular resistance and capacitance. Abrupt changes in lung volume may lead to cardiac compression within the mediastinum, potentially causing relevant hemodynamic alterations associated with worsened clinical outcomes [35]. The evaluation of respiratory mechanics was based on ΔP and Cstat, which represent routinely available bedside variables. However, the absence of transpulmonary pressure measurements prevented a more precise separation between lung and chest wall mechanics.

In clinical practice, reductions in arterial pressure typically occur with abrupt PEEP changes. In our study, the PEEP levels applied during the intervention were low and closely spaced. The absence of statistically significant changes in arterial pressure across the tested PEEP levels should be interpreted with caution, since the hemodynamic response to PEEP is highly dependent on factors such as intravascular volume status and cardiovascular reserve. In addition, arterial pressure alone represents a macrohemodynamic variable and does not necessarily reflect changes in cardiac output or global perfusion, which were not directly measured in this study.

Analysis of PaO_2_ revealed lower values at the lowest PEEP level applied during the intervention. Increasing PEEP is a common strategy to raise PaO_2_ and, consequently, improve the oxygenation index. This approach is primarily employed in patients with established ARDS [36,37,38]. Accordingly, critical care teams often maintain this intervention in such patients. The literature thus provides stronger support for interpreting the association between PEEP and PaO_2_ in the context of ARDS [11,39,40]. However, there is limited information regarding the effects of PEEP in patients without chronic lung disease. One study demonstrated that PEEP titrated to achieve the lowest ΔP minimized lung injury in patients without chronic lung disease, although no association with intubation-related mortality was observed [41].

The interaction between PEEP and cardiovascular function is a well-recognized in mechanically ventilated patients [20]. Increased intrathoracic pressure induced by PEEP may influence venous return, right ventricular preload, and pulmonary vascular resistance, potentially leading to reductions in cardiac output depending on volume status and cardiopulmonary conditions [20]. However, these effects are complex and depend on respiratory mechanics and underlying cardiac function [20].

In our study, the evaluation of hemodynamic effects was limited to macrohemodynamic parameters, as these variables were consistently available in the dataset and represent routinely monitored variables in many intensive care settings. Advanced hemodynamic monitoring or microcirculatory assessments were not systematically collected and therefore could not be analyzed. Consequently, although the potential hemodynamic effects of PEEP should be acknowledged when interpreting ventilatory parameters, a detailed evaluation of cardiopulmonary interactions was beyond the scope of the present investigation and should be explored in future studies using dedicated hemodynamic monitoring strategies.

The hemodynamic response to PEEP may be influenced by several physiological factors, including volemic status and ventricular function. Dynamic indices of preload responsiveness, such as pulse pressure variation, were not systematically collected in the present study and therefore could not be included into the analysis.

The present study aimed to evaluate the impact of PEEP on macrohemodynamics, gas exchange, and ΔP in patients with no documented prior lung disease. Therefore, higher PEEP levels than those applied in the intervention were deemed unnecessary. Based on our findings, clinical staff may safely use lower PEEP levels during MV without adversely affecting the evaluated markers.

Our study observed modest improvements in gas exchange with incremental PEEP levels, although these changes were not statistically significant. Previous studies evaluating PEEP increments during surgery reported improvements in gas exchange; however, these studies did not assess whether such improvements persisted into the postoperative period [25,31,42]. In those studies, lung recruitment maneuvers were performed because data were collected immediately after intubation. In contrast, our intervention was conducted during the early phase of IMV.

According to the literature, decremental PEEP titration is typically performed after a recruitment maneuver and is used to identify the PEEP level associated with optimal respiratory-system compliance, with the goal of maintaining lung recruitment [43]. However, the aim of our study was not to determine the optimal PEEP for patient management during MV. Rather, we sought to evaluate the impact of different PEEP levels on oxygenation, ΔP, and macrohemodynamics.

The intervention in our study did not produce significant changes in ΔP or oxygenation indices across different PEEP levels. Driving pressure (ΔP) is recognized as a reliable bedside variable for predicting VILI, mortality, and oxygenation outcomes. However, its impact on oxygenation occurs only when higher PEEP levels lead to a reduction in ΔP and consequent optimization of gas exchange. Moreover, changes in ΔP following PEEP adjustment can provide insights into alveolar recruitment potential: an increase in ΔP with higher PEEP suggests low recruitability and indicates that the patient is unlikely to benefit from further PEEP increments [12,13,44,45,46,47]. In this context, our findings support the use of lower PEEP levels when ΔP remains unchanged across different settings, as this strategy may protect the alveoli from injury. By avoiding unnecessary increases in PEEP, we can minimize the mechanical stress associated with repetitive alveolar opening and closing, thereby reducing the risk of VILI. Furthermore, clinical decision-making should be geared towards personalized bedside care, integrating clinical status, comorbidities, and the evolution of pulmonary mechanics, rather than a purely numerical and rigid interpretation.

Taken together, the present findings reinforce the physiological and clinical relevance of systematically evaluating moderate PEEP adjustments in a population that is frequently exposed to IMV but remains underrepresented in dedicated physiological studies. Most of the currently available evidence on PEEP titration derives from cohorts with acute lung injury or ARDS, in whom recruitment potential, compliance behavior, and risk–benefit relationships differ substantially. By focusing on patients with preserved respiratory mechanics, the present study addresses a relevant knowledge gap and provides data that are directly applicable to a common clinical scenario in routine intensive care practice, where PEEP selection is often empirical.

The methodological structure of the study further strengthens the interpretability of the results. The crossover intra-individual design allowed each participant to serve as their own control, thereby minimizing interindividual variability and increasing internal validity when comparing physiological responses across different PEEP levels. In parallel, all other ventilatory parameters were intentionally maintained constant throughout the protocol, ensuring that the observed changes in macrohemodynamics, gas exchange, and ΔP can be more confidently attributed to PEEP variation itself rather than to concurrent ventilatory adjustments. This level of ventilatory control, combined with protocolized stepwise PEEP changes and standardized measurement timing, represents an important methodological advantage and enhances the robustness of the physiological inferences.

In addition, the interpretation of these findings should be framed within the recognized interaction between PEEP and global respiratory system compliance, which reflects the combined mechanical behavior of the lung and chest wall. The physiological response to PEEP is known to vary according to factors such as chest wall stiffness, obesity, and abdominal pressure. By excluding patients with overt structural lung disease and by studying a cohort without a high prevalence of conditions that markedly distort chest wall mechanics, the present results predominantly reflect PEEP behavior in individuals with preserved global compliance. This targeted approach improves conceptual clarity and supports the principle that PEEP titration should be individualized according to baseline mechanical characteristics rather than uniformly extrapolated from injured-lung populations.

Moreover, because the data were obtained under real-world ICU conditions within a university hospital environment, the results maintain practical clinical relevance while preserving physiological rigor. The combination of a clearly defined and understudied population, strict ventilatory control, intra-individual comparison, and comprehensive physiological assessment constitutes a central strength of this work. Collectively, these elements contribute novel and clinically meaningful information regarding the effects of moderate PEEP levels in patients with preserved lung mechanics and support more physiology-driven approaches to ventilatory management in this group.

The main limitations of our study were the relatively small sample size and the use of closely spaced PEEP levels during the intervention. Additionally, we were unable to collect certain patient characteristics (e.g., severity scores) due to incomplete information in the medical records. Patients with preexisting lung disease or hypoxemia were also not included, although these conditions are precisely those in which PEEP might exert greater effects. We recognize that underlying subclinical lung disease cannot be definitively ruled out, which is a common challenge in research. In this context, future studies should evaluate similar interventions primarily in patients with hypoxemia. Our cohort was characterized by heterogeneous clinical profiles that, although representative of routine care at the University Hospital, limit the ability to elucidate specific physiological mechanisms underlying respiratory function and PEEP responsiveness. In trauma patients, pulmonary contusions may evolve during the first 24–48 h after injury. Therefore, although patients with clinical evidence of pulmonary injury were excluded from the study, late pulmonary changes cannot be completely ruled out.

We acknowledge that advanced hemodynamic monitoring was not performed, which limits a more comprehensive characterization of patients’ circulatory status and may have reduced the sensitivity for detecting subtle hemodynamic effects associated with PEEP adjustments. Furthermore, our study evaluated only the short-term physiological effects of varying PEEP levels; therefore, the findings should not be extrapolated to long-term outcomes or interpreted as evidence of clinical safety. Also, future studies should investigate whether the type of elective surgery acts as an independent variable in this clinical setting.

The study evaluated the physiological response to PEEP using routinely monitored macrohemodynamic parameters rather than advanced hemodynamic monitoring. Therefore, potential effects of PEEP on cardiac output or detailed cardiopulmonary interactions could not be fully assessed and should be interpreted with caution. Additional perfusion markers such as central venous oxygen saturation and the venous–arterial CO_2_ gap were not systematically collected. These parameters could provide further insight into tissue oxygen delivery and perfusion during PEEP adjustments and should be considered in future investigations. Hemodynamic assessment was limited to routinely monitored macrohemodynamic parameters obtained from IBP monitoring; therefore, these variables should not be interpreted as direct markers of cardiac output or ventricular function.

In Brazil, there is considerable variability in standards of care across ICUs [48], which may influence clinical practices and patient outcomes. Therefore, future multicenter studies should be conducted to better reflect real-world settings, similar to large collaborative initiatives promoted by National Institute for Health and Care Research during the coronavirus disease 2019 (COVID-19) pandemic [49,50,51,52].

## 5. Conclusions

In patients with no documented prior lung disease requiring IMV, we observed that moderate PEEP levels within the tested range were not associated with significant changes in macrohemodynamics, gas exchange, or ΔP. These findings indicate that, under the controlled conditions of the present protocol, incremental adjustments within this moderate PEEP range were not associated with relevant short-term physiological alterations in the evaluated parameters.

Despite the heterogeneity of the study population, which should be recognized as a relevant limitation and a potential source of variability in physiological responses, our findings provide insights into macrohemodynamic and respiratory responses to moderate PEEP adjustments in a case mix representative of general ICUs. This pragmatic profile enhances the external applicability of the results while reinforcing that the observed effects should be interpreted within the specific physiological context studied.

Importantly, these findings should not be interpreted as defining optimal PEEP levels or replacing individualized PEEP titration. The optimal level of PEEP remains dependent on respiratory system mechanics and patient-specific factors, including chest wall mechanics and clinical conditions. Within the range evaluated in this study, the absence of significant physiological changes suggests that moderate PEEP adjustments may be tolerated without major alterations in the assessed parameters.

Nevertheless, these results should be interpreted with caution, given the study’s limitations, including the relatively small sample size and the heterogeneity of patient characteristics, which reflect the complexity of routine care in a university hospital setting. Larger randomized controlled trials are warranted to validate these observations and to further clarify the physiological effects of PEEP titration in patients without preexisting lung disease.

## Figures and Tables

**Table 1:** Clinical and demographic characteristics of the patients included in the study.

Markers	Data	Hospital Discharge (70; 46.7%)	Death (80; 53.3%)	N (%)	*p*-Value
Sex	Female	28 (40.0%)	25 (31.2%)	53 (35.3%)	0.306 *
	Male	42 (60.0%)	55 (68.8%)	97 (64.7%)	
Age (years)		45.60 ± 19.42	61.04 ± 15.72	53.75 ± 19.13	<0.001 ***
Body mass index (kg/m^2^)		24.67 ± 3.90	24.88 ± 3.77	24.78 ± 3.82	0.763 ***
Primary diagnosis at intensive care unit admission	Traumatic brain injury	18 (25.7%)	12 (15.0%)	30 (20.0%)	0.151 *
	Polytrauma	27 (38.6%)	10 (12.5%)	37 (24.7%)	<0.001 *
	Non-pulmonary sepsis ^a^	10 (14.3%)	13 (16.3%)	23 (15.3%)	0.822 *
	Elective surgical procedures	19 (27.1%)	22 (27.5%)	41 (27.3%)	1.000 *
	Acute myocardial infarction	0 (0.0%)	2 (2.5%)	2 (1.3%)	0.499 **
	Cerebrovascular accident (ischemic or hemorrhagic stroke)	7 (10.0%)	8 (10.0%)	15 (10%)	1.000 *
	Subarachnoid hemorrhage	4 (5.7%)	10 (12.5%)	14 (9.3%)	0.173 **
	Type 1 and type 2 diabetes mellitus	2 (2.9%)	1 (1.3%)	3 (2.0%)	0.599 **
	Neurological and psychiatric disorders	8 (11.4%)	9 (11.3%)	17 (11.3%)	1.000 *
	Cardiopathies (including structural and ischemic heart disease)	5 (7.1%)	11 (13.8%)	16 (10.7%)	0.289 *
	Motor sequelae	0 (0.0%)	1 (1.3%)	1 (0.7%)	1.000 **
	Other	1 (1.4%)	5 (6.3%)	6 (4.0%)	0.216 *
Patient origin	Surgical	50 (71.4%)	48 (60.0%)	98 (65.3%)	0.170 *
	Clinical	20 (28.6%)	32 (40.0%)	52 (34.7%)	
Ventilator-associated pneumonia (during intensive care unit stay) ^b^	Present	22 (31.4%)	25 (31.3%)	47 (31.1%)	1.000 *
	Absent	48 (68.6%)	55 (68.8%)	103 (68.7%)	
Requirement for tracheostomy	Yes	34 (48.6%)	25 (31.3%)	59 (39.3%)	0.044 *
	No	36 (51.4%)	55 (68.8%)	91 (60.7%)	
Comorbidities	Diabetes mellitus	11 (15.7%)	21 (26.3%)	32 (21.3%)	0.162 *
	Systemic arterial hypertension	18 (25.7%)	34 (42.5%)	52 (34.7%)	0.039 *
	Tobacco use (smoking)	11 (15.7%)	16 (20.0%)	27 (18.0%)	0.530 *
	Alcohol consumption	11 (15.7%)	14 (17.5%)	25 (16.7%)	0.829 *
	Substance use disorder (drug addiction)	6 (8.6%)	7 (8.8%)	13 (8.7%)	1.000 *
	Dyslipidemia	5 (7.1%)	5 (5.0%)	9 (6.0%)	0.734 **
	Cardiopathy	9 (12.9%)	18 (22.5%)	27 (18.0%)	0.141 *
	Other comorbidities	20 (28.6%)	26 (32.5%)	43 (35.3%)	0.603 *
Types of ventilation	Pressure-controlled ventilation	7 (10.0%	5 (6.3%)	12 (8.0%)	0.293 *
	Volume-controlled ventilation	63 (90.0%)	75 (93.8%)	138 (92.0%)	
Length of hospital stay (days)		27.19 ± 15.68	19.17 ± 16.35	22.90 ± 16.50	<0.001 ***
Duration of mechanical ventilation (days)		15.32 ± 11.89	13.12 ± 10.69	14.16 ± 11.29	0.298 ***

Statistical analyses were performed using the chi-square test (*), Fisher’s exact test (**), and the Mann–Whitney U test (***). A two-sided alpha level of 0.05 was considered statistically significant, and *p*-values < 0.05) are reported in bold. Descriptive statistics are presented as mean ± standard deviation for continuous variables and as absolute and relative frequencies (%, percentage) for categorical variables. ^a^: Patients with sepsis of pulmonary origin recorded as the reason for hospitalization were not recruited. ^b^: All participants were enrolled within the first 24 h of initiation of mechanical ventilation and had no diagnosis of ventilator-associated pneumonia at baseline. Ventilator-associated pneumonia was defined according to standard criteria as pneumonia occurring ≥ 48 h after the start of mechanical ventilation and was recorded only when diagnosed during the intensive care unit stay after study inclusion.

**Table 2:** Association between positive end-expiratory pressure (PEEP) levels and hemodynamics, gas exchange, and driving pressure (ΔP) in intubated patients at the University Hospital.

Marker	PEEP 6	PEEP 8	PEEP 10	*p*-Value
Systolic arterial pressure (mmHg)	129.25 ± 26.64	129.01 ± 27.04	126.43 ± 25.45	0.042 *
Diastolic arterial pressure (mmHg)	63.63 ± 12.20	65.56 ± 13.64	63.87 ± 11.69	0.153
Mean arterial pressure (mmHg)	85.39 ± 14.51	85.85 ± 15.23	84.25 ± 14.52	0.029 *
Peripheral oxygen saturation (SaO_2_, %)	97.79 ± 2.29	97.73 ± 2.31	97.78 ± 2.22	0.649
Arterial oxygen partial pressure (PaO_2_, mmHg)	121.39 ± 36.96	118.97 ± 34.49	120.47 ± 38.11	0.716
Arterial carbon dioxide partial pressure (PaCO_2_, mmHg)	41.62 ± 6.87	41.87 ± 6.78	42.54 ± 6.45	0.073
Oxygenation index (PaO_2_/FiO_2_ ratio)	373.16 ± 140.50	364.90 ± 127.60	365.27 ± 128.46	0.844
ΔP (cmH_2_O)	10.13 ± 3.19	9.89 ± 3.11	9.99 ± 3.44	0.415
Static compliance (Cstat, mL/cmH_2_O)	46.80 ± 17.75	48.30 ± 20.16	49.32 ± 23.50	0.415

FiO_2_, fraction of inspired oxygen. The following dependent variables were included: respiratory and hemodynamic parameters, including PaO_2_, PaCO_2_, SaO_2_, oxygenation index, arterial pressures, ΔP, and Cstat. Inference analyses were performed using the Friedman test to evaluate differences in dependent variables across multiple PEEP levels. A two-sided alpha level of 0.05 was considered statistically significant, and *p*-values < 0.05 are reported in bold. *: *p*-values were significant after adjustment for multiple comparisons using the Bonferroni correction method for the comparison between PEEP levels of 6 and 10 cmH_2_O.

**Table 3:** Association between positive end-expiratory pressure (PEEP) levels and hemodynamics, gas exchange, and driving pressure (ΔP) stratified by hospital mortality in intubated patients at the University Hospital.

Marker	Outcomes	PEEP 6	PEEP 8	PEEP 10	*p*-Value
Systolic arterial pressure (mmHg)	Hospital discharge	129.24 ± 24.85	127.77 ± 23.93	125.40 ± 20.53	0.047 *
	Death	129.25 ± 28.27	130.09 ± 29.60	127.33 ± 29.18	0.374
		0.818	0.708	0.753	
Diastolic arterial pressure (mmHg)	Hospital discharge	64.13 ± 12.70	64.73 ± 12.02	63.94 ± 11.13	0.608
	Death	63.19 ± 11.81	66.29 ± 14.96	63.81 ± 12.23	0.051
		0.699	0.733	0.894	
Mean arterial pressure (mmHg)	Hospital discharge	85.49 ± 13.82	84.69 ± 12.97	83.51 ± 11.48	0.017 *
	Death	85.30 ± 15.18	86.88 ± 16.97	84.89 ± 16.78	0.177
		0.917	0.568	0.938	
Peripheral oxygen saturation (SaO_2_, %)	Hospital discharge	97.84 ± 2.29	97.84 ± 2.06	97.87 ± 1.86	0.572
	Death	97.74 ± 2.30	97.63 ± 2.51	97.70 ± 2.50	0.885
		0.715	0.832	0.887	
Arterial oxygen partial pressure (PaO_2_, mmHg)	Hospital discharge	120.40 ± 32.30	116.61 ± 28.59	115.76 ± 29.70	0.718
	Death	122.26 ± 40.80	121.03 ± 39.00	124.60 ± 43.94	0.653
		0.804	0.959	0.432	
Arterial carbon dioxide partial pressure (PaCO_2_, mmHg)	Hospital discharge	41.43 ± 6.72	41.69 ± 6.11	42.86 ± 6.01	0.025 *
	Death	41.79 ± 7.04	42.04 ± 7.35	42.26 ± 6.84	0.800
		0.741	0.798	0.624	
Oxygenation index (PaO_2_/FiO_2_ ratio)	Hospital discharge	386.97 ± 143.60	374.49 ± 125.39	370.20 ± 124.94	0.697
	Death	361.08 ± 137.48	356.52 ± 129.71	360.95 ± 132.09	0.725
		0.215	0.251	0.452	
Driving pressure (ΔP, cmH_2_O)	Hospital discharge	9.81 ± 2.80	9.69 ± 3.06	9.86 ± 3.06	0.748
	Death	10.41 ± 3.50	10.06 ± 3.16	10.11 ± 3.58	0.432
		0.338	0.251	0.452	
Static compliance (Cstat, mL/cmH_2_O)	Hospital discharge	47.51 ± 16.53	48.64 ± 18.00	49.25 ± 21.71	0.748
	Death	46.18 ± 18.83	47.99 ± 21.99	49.39 ± 25.09	0.432
		0.340	0.340	0.636	

FiO_2_, fraction of inspired oxygen. Different PEEP levels were considered as within-subject factors. The following dependent variables were included: respiratory and hemodynamic parameters, including PaO_2_, PaCO_2_, SaO_2_, oxygenation index, arterial pressures, ΔP, and Cstat. Clinical outcome (death or hospital discharge) was included as a between-subject factor. Inference analyses were performed using the Friedman test to evaluate differences in dependent variables across multiple PEEP levels, accounting for outcome groupings. Additionally, the Mann–Whitney U test was used to compare differences between two independent groups for the dependent variables. A two-sided alpha level of 0.05 was considered statistically significant, and *p*-values < 0.05 are reported in bold. *: *p*-values were not significant after adjustment for multiple comparisons using the false discovery rate (FDR) method (*p*-values > 0.05).

## Data Availability

The original contributions presented in this study are included in the article material. Further inquiries can be directed to the corresponding author.

## Abbreviations

The following abbreviations are used in this manuscript:

±	Plus or minus
%	Percentage
<	Less than
>	Greater than
≤	Less than or equal to
≥	Greater than or equal to
α	Alpha
ΔP	Driving pressure
ARDS	Acute respiratory distress syndrome
BMI	Body mass index
b.p.m.	Beats per minute
cmH_2_O	Centimeters of water
cm	Centimeters
CO_2_	Carbon dioxide
Cstat	Static compliance
DP	Driving pressure
FDR	False discovery rate
FiO_2_	Fraction of inspired oxygen
g/dL	Grams per deciliter
kg/m^2^	kilograms per square meter
ICU	Intensive care unit
IMV	Invasive mechanical ventilation
IBP	Invasive blood pressure
IBM Corp.	International Business Machines Corporation
mmHg	Millimeters of mercury
mL/kg	Milliliters per kilogram
MV	Mechanical ventilation
N	Number of participants
PaCO_2_	Partial pressure of carbon dioxide
PaO_2_	Partial pressure of oxygen
PEEP	Positive end-expiratory pressure
pH	Potential of hydrogen
Pplat	Plateau pressure
Q-Q	Quantile–Quantile
SaO_2_	Arterial oxygen saturation
SPSS	Statistical Package for the Social Sciences
USF	São Francisco University (Universidade São Francisco)
VILI	Ventilator-induced lung injury
